# Development and Validation of the Questionnaire of Academic Stress in Secondary Education: Structure, Reliability and Nomological Validity

**DOI:** 10.3390/ijerph15092023

**Published:** 2018-09-16

**Authors:** Rafael García-Ros, Francisco Pérez-González, José M. Tomás

**Affiliations:** 1 Department of Developmental and Educational Psychology, University of Valencia, 46010 Valencia, Spain; francisco.perez-gonzalez@uv.es; 2 Department of Methodology for the Behavioral Sciences, Advanced Research Methods Applied to Quality of Life Promotion—ARMAQoL, University of Valencia, 46010 Valencia, Spain; Jose.M.Tomas@uv.es

**Keywords:** academic stress, psychological and physical well-being, adolescence, secondary education, validity, reliability, gender and age differences

## Abstract

This study presents the validation process of the Questionnaire on Academic Stress in Secondary Education (QASSE) designed to assess the wide variety of school sources and situations related to academic stress in adolescence, and their relationship with students’ physical and psychological well-being. The participants were 860 Spanish high school students (52.9% girls) with an average age of 14.62 years (SD = 1.8). Through a cross-validation process, results supported the QASSE multifactorial structure with four first-order factors—academic overload, interaction with classmates, family pressure, and future-oriented perspective—and a second-order factor of academic stress, showing a significant and intense relationship with adolescents’ psychological and physical well-being. Results also highlight the effects of the gender and educational level interaction on the students’ stress, with girls showing higher levels of stress in the transition courses between educational phases (sophomore and junior years). The QASSE demonstrates good validity and reliability, showing potential for both research and educational application. The results show the high impact of the QASSE dimensions on psychological and physical well-being in adolescence, highlighting its special usefulness for designing and adjusting educational prevention and intervention actions in this area to the students’ specific characteristics and needs.

## 1. Introduction

Academic stress is a widespread phenomenon in the different stages of the educational system, and it adversely affects students’ personal, emotional, and physical well-being [1,2,3,4,5,6], as well as their learning and performance levels [7,8]. Different studies also highlight its relationship with early school dropout [9] and internalizing and externalizing problems in school contexts [10,11]. Academic stress is especially relevant in adolescence because the school environment is one of the most significant life contexts in this developmental stage and one of the sources of stress most identified by adolescents [12,13]. In addition, transitions between educational stages are usually related to higher levels of stress [7,14]. They can have a negative influence on students’ academic, personal, and social adjustment, and their levels of self-esteem and achievement [15].

In spite of its demonstrated prevalence and relevance in adolescence, various authors point out serious gaps and problems in the assessment of academic stress in this developmental stage. Some of the most important shortcomings are (a) dissatisfaction with the assessment instruments currently available [16] and (b) the low number of studies focused on secondary education, compared to university level education, and inconsistencies in their conclusions about the relationships among gender, educational level, and academic stress [17,18].

Focusing on the first question, despite several self-report instruments having been developed to assess academic stress, recent studies refer to their limitations [11,16,19]—especially those developed in Spanish [16,19]—alleging that they (a) are generic in nature and decontextualized from the school setting, (b) have a one-dimensional nature and provide little information for intervention in this area, and (c) focus on partial aspects of academic stress. Thus, several authors emphasize that few instruments consider the broad range of potentially stress-producing academic conditions and that there is a need for instruments with contrasted validity and reliability [11]. Noteworthy shortcomings are identified in the validation of the available instruments, such as not using confirmatory techniques to show the consistency and stability of their structure across different samples [20]. Among the few exceptions, recent studies [21,22] highlight the Spanish adaptation of the Student Stress Inventory Manifestation, which evaluates the different manifestations (physiological, emotional, and behavioral) of academic stress in adolescence.

Focusing on the second question, the inconsistencies among the results of previous studies that analyze the possible effects of adolescents’ gender and educational level on their levels of academic stress make this question an especially relevant objective in this study. Thus, although most studies point out that girls express greater academic stress than boys [11,23], some recent evidence revealed that adolescents’ perceptions of school-related stressors are similar in both genders [3,23], or that girls present greater stress when faced with some types of stressors—e.g., related to worries about school achievement—whereas boys present greater stress related to others—e.g., conflicts with parents and/or teachers—[4,5,24]. Moreover, studies that analyze this question in transitions between educational stages suggest that girls show higher levels of stress during these periods [23,25,26]. Thus, in spite of the large volume of studies that analyze stress in adolescents in different life contexts, these results highlight that possible differences in academic stress depending on adolescents’ gender and educational level are still not clear [27]. These questions are especially relevant, given that they limit and impede the design and evaluation of prevention and intervention programs in the school setting, and their adaptation to students’ specific needs [28].

All these considerations underscore the need to develop and validate an academic stress assessment instrument in adolescence that considers the broad range of elements that make up the school context, can easily be applied in schools, provides valid and reliable measures of their evolution in this context, and helps the professionals involved to relate assessment and intervention.

### Objectives

Thus, the purpose of this study is to design and validate an instrument to assess academic stress in adolescence, analyzing the effects of students’ educational level and gender on its underlying dimensions. More specifically, the study objectives are:

(a) To analyze the psychometric properties of the Questionnaire on Academic Stress in Secondary Education, hereinafter QASSE.
(b) To determine the relationship between the dimensions of the QASSE and students’ physical and psychological well-being as part of its nomological validity net.
(c) To analyze the relationships among academic stress, educational level, and gender in secondary education (12–18 years).

## 2. Materials and Methods

### 2.1. Item Development

The development of the initial pool of items on the QASSE was based on a large number of previous studies focused on analyzing students’ everyday stressors in the school context, such as schoolwork pressure, school/leisure conflicts, worries about school achievement, difficulties with peers at school, conflicts with teachers and parents, and concerns about the future and their effects on adolescents’ personal well-being [3,5,13,25,27]. Three experienced researchers in adolescent developmental and educational psychology and three school psychologists from different public secondary schools participated. A comprehensive and exhaustive initial pool of 30 items was developed. The items were originally written in Spanish. These items were related to potentially stress-producing situations in the school context, and special care was taken to ensure that their wording was simple and accessible to adolescents [29,30].

The 30-item draft was submitted and administered to six secondary school teachers and 18 secondary students (12 from compulsory secondary education and six from post-compulsory secondary education), using a Likert-type response scale from 1 (very low) to 5 (very high). Feedback obtained from the teachers and students emphasized the relevance and applicability of all the items, and minor adjustments were made in the wording of various items in order to make the vocabulary more easily understood by adolescents.

Thus, the initial version of the QASSE used in the study comprised 30 items related to different potentially stress-producing situations in secondary education, see Table 1. Students’ responses reveal their stress level in the different school situations on a Likert-type scale with five response options (1 = “Very low”, to 5 = “Very high”).

### 2.2. Participants

Participants in the study were 860 high school students in seven public high schools in a large city in Eastern Spain during the 2014–2015 academic year. Their mean age was 14.62 years (SD = 1.8). Of all the participants, 70.2% were studying Compulsory Secondary Education (7th grade, *n* = 132; 8th grade, *n* = 150; 9th grade, *n* = 154; 10th grade, *n* = 168; total Compulsory Secondary Education, *n* = 604), whereas 29.8% (*n* = 256) were studying Post-Compulsory Secondary Education (pre-university) (11th grade, *n* = 150; 12th grade, *n* = 106; All post-compulsory secondary education, *n* = 256). All the participating schools are located in areas with a low-medium socioeconomic level. Data were collected among all grade levels at all schools.

### 2.3. Measures

Other measures were used in this study to analyze the validity of the QASSE. The measures employed were chosen as they may be considered part of the nomological net in the sense of Cronbach and Meehl [31], and therefore offer evidence on nomological validity. To analyze the convergent validity of the QASSE, the Spanish adaptations of the *General Health Questionnaire* -GHQ-12- [32,33] and the *List of Somatic Complaints* -LQS- [34] were administered.

General Health Questionnaire 12-items (GHQ-12). The GHQ-12 objective is to evaluate the general mental health or current well-being at the level of the general population. It contains twelve items with a four-level response scale (0 = “Less than usual”; 3 = “A lot more than usual”); six items are expressed in terms of clinical symptomatology, and the rest are worded in a positive way. The response values of the positive items were inverted to obtain an estimation of the degree of severity of the absence of mental health. The scale’s three underlying factors [35] corresponding to Anxiety and Depression, Social Dysfunction, and Loss of Self-esteem, showed adequate internal consistency (Cronbach’s alpha of 0.75, 0.81, and 0.70, respectively).

LQS. List of Somatic Complaints. The LQS objective is to identify the frequency with which children and adolescents experience and feel pain. It consists of eleven items related to the frequency with which they experienced different physical complaints in recent weeks (e.g., “stomachache”), using a scale with three response options (1 = “Never”; 3 = “Often”). Its internal consistency in this study was 0.84.

### 2.4. Procedure

The study was reviewed and approved by the Ethics Committee of the University of Valencia (code number H1523870265031). Likewise, it had authorization from the Board of Education of the Valencian Government to access the schools and to develop the study. After obtaining the informed consent of the schools and families of the participants, the students filled out the instruments collectively and voluntarily during school hours the week before the first semester exams. The instruments were administered by collaborating psychologists from the research team in a 50-minute session. In order to perform a cross-validation of the QASSE, the resulting database was randomly divided in half. The first half (sample 1) was used for exploratory purposes, and the second half (sample 2) was used for confirmatory ends.

### 2.5. Analysis

Sample 1 responses were submitted for principal components analysis (PCA) with oblimin rotation using SPSS 22 (SPSS Inc., Chicago, IL, USA). Dimensions with values greater than 1.5 were selected, considering a factorial saturation greater than 0.40 in only one dimension as the criterion for selection and assignment of the factors. To test the validity of the structure resulting from the PCA, the sample 2 responses were analyzed with a confirmatory factor analysis (CFA) specified and estimated in Mplus (Muthén & Muthén, Los Angeles, CA, USA) [36]. A weighted Least Square Mean and Variance Corrected (WLSMV) method of estimation was employed in order to accommodate the non-normality and ordinal nature of the indicators in the first set of CFA models (those analyzing the items) and Robust Maximum Likelihood (MLR) in the model testing for the nomological validity of the scale [37]. We assessed model fit using the chi-square statistic, the Comparative Fit Index (CFI), and the Root Mean Square Error of Approximation (RMSEA)—the indices available for this type of estimation. We used the following criteria to determine good fit: CFI above 0.90 (better if above 0.95) and RMSEA below 0.08 [38]. In addition to overall fit indexes, the acceptability of the model was evaluated by the strength and interpretability of the parameter estimates and the absence of large and substantively meaningful modification indices with Weighted Least Square Mean and Variance Corrected. The reliability (internal consistency) of the resulting subscales was estimated with alpha coefficients, Average Extracted Variance (AVE), and Composite Reliability Indexes (CRI).

To analyze the nomological validity of the QASSE scores, a new structural model was specified and submitted for evaluation through CFA, considering the dimensions underlying the QASSE, the GHQ-12, and the LQS. Given the sample size and the large number of indicators that make up the three scales, the decision was made to parcel the items from the QASSE and the LQS and consider the scores on the subscales of the GHQ-12 as indicators of psychological well-being. Establishing parcels of items produces more stable solutions, better fit levels, less bias, and lower estimation errors [39].

Finally, considering all the participants in the study, a factorial Multivariate Analysis of Variance (MANOVA) was used to analyze the possible effects of the students’ educational level and gender on their academic stress levels. MANOVA was calculated in SPSS 22.

## 3. Results

### 3.1. Principal Components Analysis

The PCA showed the existence of four underlying dimensions in the initial version of the QASSE. Table 1 shows the eigenvalues, percentage of variance explained by the retained factors, means and standard deviations of the items in that factor, and their alphas. Table 2 presents the basic descriptors and communalities of the 30 initial items, as well as the factor loadings in the different dimensions, which together explain 50.4% of the total variance in the data.

Based on the items with the greatest saturation in factor 1 (“Taking exams”, “Academic overload—having too many exams and tasks to do—”, and “Lack of time to fulfill all the activities we are asked to do”), this factor was called Academic Overload and School Performance. Given the most representative items in factor 2 (“My relationships with my classmates”, “Working with classmates on tasks in class”, and “Intervening in class—e.g., asking questions, participating in debates—”), this factor was labeled Interaction with Classmates. Factor 3 was called Family Pressure, based on its most representative items (“Family discussions and conflicts caused by my studies”, “The fact that my parents are always on top of me—e.g., whether I do my homework and activities, my grades, …—”, and “Family pressure to obtain good grades”). The last factor was called Future Perspectives because all the items included in it refer to this question (e.g., “Choosing subjects in the coming courses”, “Getting or keeping a grant to study”, “Future academic and professional perspectives”).

Six items were ruled out in later analyses. Five of them showed saturations above 0.40 in more than one dimension (items 8, 13, 11, 15, and 21), and one (item 2) did not reach the minimum saturation considered. After their elimination, the reliability of the scale overall was 0.92, which suggests considering a global score of academic stress. 

### 3.2. Confirmatory Factor Analysis

**Figure 1:**
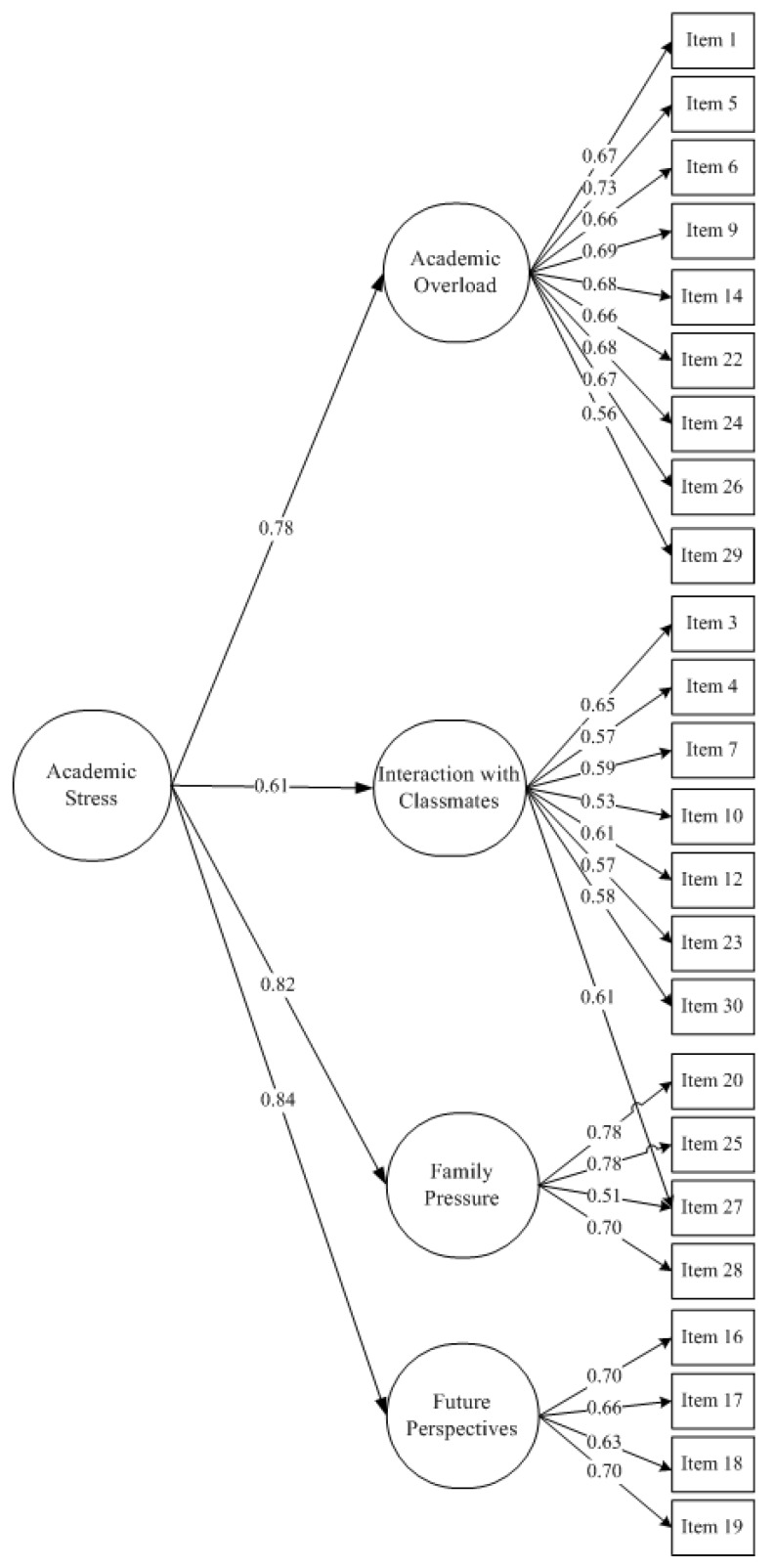
Standardized factor loadings for the M3r# model.

To determine the structural validity of the solution derived from the PCA with sample 1, various CFAs were carried out with the sample 2 responses, see Table 3. Initially, three alternative structural models for the QASSE were considered: A one-dimensional model (M1); a model stemming from the preceding PCA with four oblique factors (M2); a third model, also derived from the previous PCA, considering a structure with four first-order oblique factors along with a second-order factor (M3). Given that the results showed the existence of a large modification index affecting item 27, a correction was made in order to improve model fit. Item 27 was cross-loaded in its original factor (factor 3) and in factor 2. Both models M2 and M3 were corrected. Goodness-of-fit indexes are in Table 3. The second order model corrected with the cross-loading offers the best trade-off between fit and parsimony and it is therefore retained as the best representation of the data. Figure 1 shows the values of the estimated parameters through the CFA, revealing that all the factorial saturations are significant and equal to or greater than 0.45.

The internal consistency estimates of both the first-order dimensions and the second-order factor from the QASSE were adequate. Alpha, AVE, and CRI were calculated for each factor. Academic Overload and school performance (factor 1) presented the highest value (*α* = 0.84, AVE = 0.45, CRI = 0.87), followed by Family Pressure (factor 3, *α* = 0.77, AVE = 0.56, CRI = 0.83), Interactions with Classmates (factor 2, *α* = 0.74, AVE = 0.44, CRI = 0.78), and Future Perspectives (factor 4, *α* = 0.73, AVE = 0.45, CRI = 0.77). The internal consistency of the general academic stress factor was *α* = 0.89.

### 3.3. Academic Stress, Personal Well-Being, and Somatic Complaints

To analyze the nomological validity of the QASSE scores with the GHQ-12 and LQS, a new structural model was evaluated. Given the large number of indicators in the instruments, different parcels of items were established in two dimensions of the QASSE and in the LQS, whereas the scores on the subscales of the GHQ-12 were considered indicators of psychological well-being. The parcels in the QASSE were established according to the elements on the subscales resulting from the PCA and CFA: Academic Overload and Interaction with Classmates were modeled by considering parcels made up of two consecutive items, with the exception of the final grouping, which contained three items; Family Pressure and Future Perspectives were modeled with their four original items to avoid having only two indicators in each. The LQS was modeled with four item parcels, each composed of three consecutive items, with the exception of the last item parcel, which had only two.

**Figure 2:**
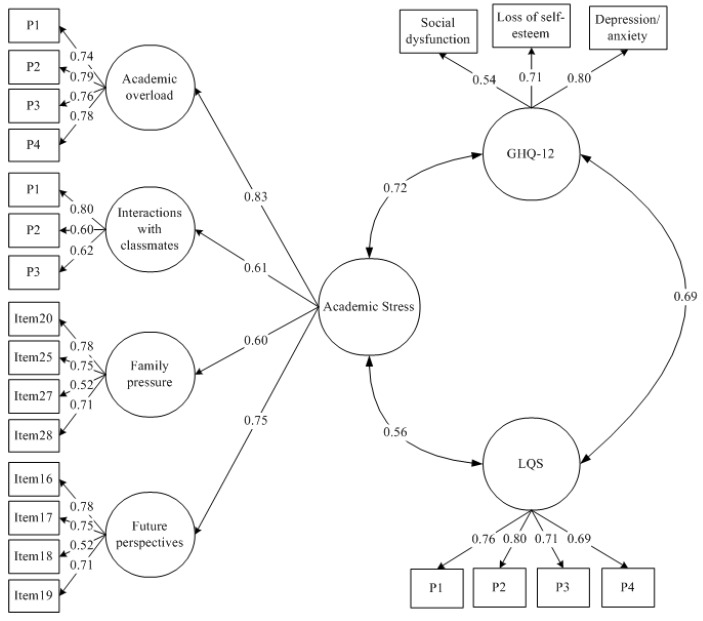
Confirmatory factor model relating QASSE, General Health Questionnaire 12-items (GHQ-12), and List of Somatic Complaints (LQS).

Figure 2 shows the results of the CFA carried out to evaluate the nomological validity of the QASSE scores. The results highlight the adequate fit of the model to the data (SB*χ*^2^ (201) = 570.38, *p* < 0.001; RMSEA = 0.054, 90% CI [0.049–0.050]; CFI = 0.922), showing that all the saturations are significant and of considerable magnitude (values between 0.54 and 0.80). The relationships between the hierarchical factor from the QASSE, personal well-being (GHQ-12), and somatic symptoms (LQS) are significant at 0.001 and have high values (0.69 and 0.72, respectively).

Table 4 presents the basic descriptors, correlations, and internal consistency of the latent variables obtained from the CFA. Both the second-order factor and the four first-order factors from the QASSE show satisfactory internal consistency levels, revealing significant relationships at 0.001 with the GHQ-12 and with the LQS.

### 3.4. Gender and Educational Level Differences

To analyze possible differences due to adolescents’ gender and educational level in the QASSE dimensions, a 2 × 2 factorial MANOVA was performed with the responses of all the participants. The results show the existence of significant effects on academic stress, based on gender, Λ = 0.95, *F* (4, 867) = 12.6, *p* < 0.001, *η*^2^ = 0.06, and educational level, Λ = 0.80, *F* (20, 2876.5) = 3.67, *p* < 0.001, *η*^2^ = 0.05, as well as the gender and educational level interaction, Λ = 0.93, *F* (20, 2876.5) = 3.03, *p* < 0.05, *η*^2^ = 0.02. Through the corresponding ANOVAS, a more detailed analysis was performed of their effects on the dimensions of the QASSE. The results shown in Table 5 show the significant effects of the gender variable on Academic overload, Future perspectives, and the second-order factor. Significant effects of educational level are also observed in all the stress dimensions. The gender and educational level interaction is significant for Academic Overload, Interaction with Classmates, Future Perspectives, and the general Academic Stress factor.

**Figure 3:**
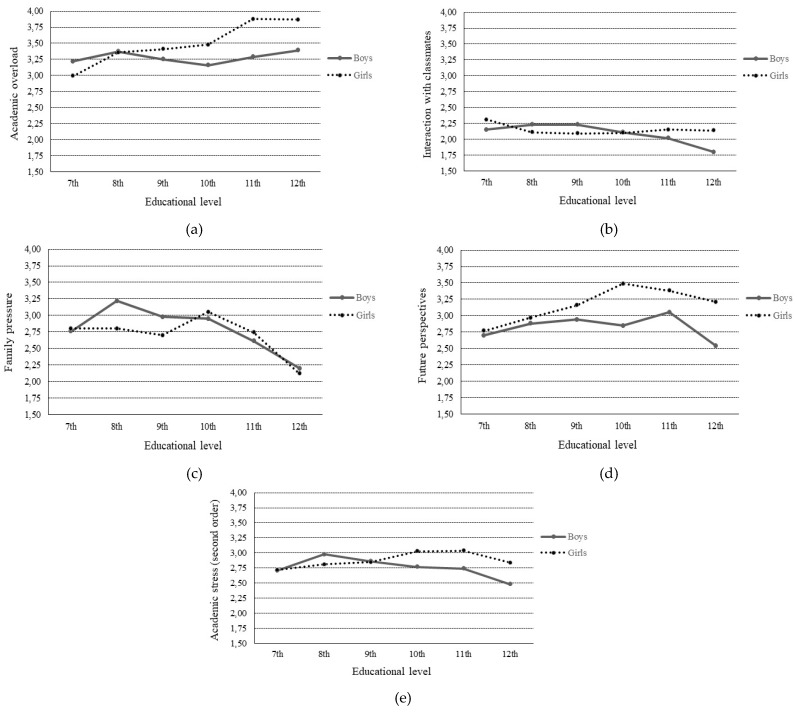
Mean scores on QASSE dimensions by gender and educational level: (a) Academic overload; (b) Interactions with classmates; (c) Family pressure; (d) Future perspectives; (e) Academic stress second order factor.

Given that the interpretation of the principal effects is subordinate to the existence of significant interactions, the comments will be based on the interactions between gender and educational level. In order to facilitate the interpretation of the results, Figure 3 shows the graphs of the average scores on the QASSE by gender and educational level.

Girls experience significantly higher levels of stress than boys on Academic Overload and school performance in the fourth year of secondary education (10th grade) (*p* < 0.01) and in both courses of upper secondary (11th and 12th grades) (*p* < 0.001), showing a strictly increasing progression in the period analyzed. By contrast, boys show homogeneous levels during the entire period.

For Interactions with Classmates, significant gender differences are only observed in 12th grade, with girls presenting higher values than boys (*p* < 0.01). The girls present homogeneous scores in the entire period analyzed, whereas the boys reduce their levels of stress. The average values reported by both genders at all levels are quite low.

In the case of Family pressure, only the main effects of educational level are significant. Girls and boys show homogeneous values until 11th grade, with a significant reduction in 12th grade (*p* < 0.01), compared to previous levels.

Although on Future Perspectives the girls present higher values at all educational levels, the differences in gender are only significant (*p* < 0.001) in the transition courses between stages—in 10th grade and 12th grade. The boys present a homogeneous profile throughout the entire period, whereas the girls show a growing progression during compulsory secondary education.

On the second-order hierarchical factor, girls show significantly higher levels of academic stress than boys in 10th grade (*p* < 0.05) and in 11th and 12th grades (*p* < 0.01). The boys present a homogeneous profile until 12th grade, whereas the girls show a growing progression between 7th grade and 11th grade.

## 4. Discussion

The main objective of this study was to present the development and validation of the QASSE, a scale for evaluating academic stress in Secondary Education. In spite of the demonstrated prevalence and relevance of academic stress in adolescence, some authors [11,16,19] point out the low number of studies devoted to this topic in this educational stage, and they have also pointed out several important shortcomings of the evaluation instruments available, limiting their usefulness for evaluation and intervention. From this perspective, this study has analyzed the dimensionality and internal consistency of the QASSE, its relationship with adolescents’ physical and psychological well-being, and the effects of students’ gender and educational level on their levels of academic stress, given the discrepancies in the conclusions from previous research [3,9,27].

### 4.1. Dimensionality and Psychometric Properties of the QASSE Scores

The results highlight that the QASSE scores provides valid and reliable information to evaluate academic stress in adolescence. More specifically, the QASSE seems especially useful for identifying levels of academic stress produced by the different types of academic stressors related to academic overload and school performance, interaction with classmates, family pressure, and future perspectives. In addition, the four subscales of the QASSE reflect dimensions that are qualitatively consistent with the conclusions of previous research on academic stress in adolescence [3,4,27], providing an instrument with contrasted validity and reliability that allows their combined assessment. We think this question is especially important in the school context because it makes it possible to evaluate and design intervention proposals for academic stress that fit students’ specific needs.

Moreover, these results emphasize the importance of considering a multidimensional perspective in the evaluation of, and intervention on academic stress, contemplating a wide variety of factors and sources found in the school context. Thus, there is a need to consider and address aspects related not only to academic overload and obtaining satisfactory outcomes, which are traditionally the ones most considered in this setting and show the highest scores in the research along with worries about the future [3,13], but also related to promoting satisfactory relationships and support among classmates [1], facing possible conflicts with the family and teachers [40],and developing effective strategies for coping with the uncertainties of their academic future [11,12].

### 4.2. Academic Stress, Psychological and Physical Well-Being of Adolescents

Consistent with the conclusions of prior research, the results highlight that all the QASSE dimensions were significantly associated with adolescents’ psychological and physical well-being [3,6,14,25]. In addition, the second-order QASSE factor also shows an inverse relationship of a large magnitude with adolescents’ psychological and physical well-being, confirming the special relevance of academic stress in this developmental period [2]. In summary, these results also highlight the importance of considering a multidimensional perspective in evaluating, preventing, and carrying out interventions on academic stress in school contexts, making it possible to respond to specific problems that students can have in this area.

### 4.3. Effects of Gender and Educational Level

The results show that the main effects of gender and educational level and the effects of the gender and educational level interaction are significant in two first-order dimensions of the QASSE (Academic overload and Future perspectives) and in the general academic stress factor. However, in the Family pressure and the Interactions whit classmates dimensions, only the main effects of educational level or the effects of gender and educational level interaction are significant, but not the main effects of gender, which some studies have found significant [4], but not others [24].

Thus, although the results converge in general terms with the conclusions of previous research, which mainly state that girls experience higher levels of stress than boys in various dimensions of academic stress, but not in others [4,7,24], and that higher levels of stress are observed in transition courses between educational stages [14], the significant gender and educational level interaction makes it possible to broaden and nuance previous results. Hence, girls mainly show higher levels of stress than boys in the transition courses between stages (10th grade and 12th grade) in the dimensions related to Academic overload, Interactions with classmates, Family pressure, Future perspectives, and the second-order factor of academic stress, which would support female adolescents’ greater vulnerability in these courses [23,25,26]. In any case, the results support the importance of paying special attention to the development of transition programs between compulsory and post-compulsory secondary education, and between the latter and university studies, because female adolescents can be especially vulnerable to the stress produced by the uncertainties and academic, personal, and social changes involved in these transitions.

Additionally, other educational implications of these results are especially remarkable in applied terms. First, the results highlight that intervention in academic stress management could be useful at all educational levels of Secondary Education, and in the different dimensions assessed by the QASSE. Second, results also highlight the QASSE’s special usefulness for designing and adjusting educational prevention and intervention actions in this area to the students’ specific characteristics and needs. On one hand, in some cases it could be especially relevant to focus the intervention on the development of effective time management skills (academic overload) and coping strategies for the evaluation situations (exams) [41,42], or in the students‘ establishment of realistic academic goals and objectives (future perspectives) [17,43]. On the other hand, given that the problems and conflicts in interpersonal relationships constitute a clear risk factor for the deterioration of performance and academic functioning [1,9], this area of intervention should also occupy a pre-eminent place in some cases, focusing the intervention on developing effective coping and problem-solving strategies in relationships with teachers, peers, and parents to obtain good results, although presumably considering intervention modalities more individualized and/or focused on the teaching staff and classroom management [6]. In any case, this issue also highlights the relevance of adjusting the intervention to the specific problems and needs evidenced for each student, without assuming that the intervention in one of the QASSE dimensions will mean progress in the others. Lastly, it’s relevant to highlight the importance of different organizational factors that can be especially relevant to reduce the academic stress that students experience, such as the coordination of the teaching staff in the assignment of tasks and in the planning for the examination calendar, the design of learning environments, and the use of teaching and assessment student-centered methodologies, or the improvement of support systems in face of difficulties that may arise in the performance of tasks and in the study of the different subjects [17,44,45].

## 5. Conclusions

In summary, this study highlights: (a) The QASSE scores provides valid and reliable information to evaluate academic stress in adolescence; (b) the importance of using a multidimensional perspective in the evaluation and intervention in academic stress in secondary education, taking into account the different agents and subsystems of the school context; (c) the close relationship between academic stress assessed by the QASSE and adolescents’ mental and physical health; and (d) the significant effects of students’ educational level and gender on academic stress levels, emphasizing the importance of planning preventive interventions in the transition courses between stages, with girls presenting higher levels of vulnerability to these transitions. The limitations of the study are related to its cross-sectional nature, the use of self-report measures of academic stress, somatic complaints and well-being, and the fact that these measures were administered at a specific time point in the academic year. Future studies will have to consider other sources of information, such as the teachers’ and parents’ perspectives, as well as behavioral variables related to students’ well-being (e.g., missing classes due to illness, requests for educational and psychological support). Moreover, it would also be especially relevant to develop longitudinal studies in order to determine the evolution of academic stress in adolescence, also considering how it varies throughout the academic course depending on the proximity of exams and their outcomes, as well as whether the proximity of exams affects the dimensions of academic stress in a different way (for example, making more salient the dimensions of academic overload or family pressure). In any case, the results reveal that the QASSE has good reliability and validity estimates and may therefore evaluate academic stress in Secondary Education, allowing greater comprehension of this phenomenon and facilitating the development of intervention proposals focused on students’ specific needs.

## Figures and Tables

**Table 1:** Eigenvalues, percentage of variance explained, average value and standard deviation of the items in each factor and internal consistencies (alphas).

Component	Eigenvalue	% of Variance	Mean	SD	Alpha
One	5.90	19.78	3.33	0.98	0.89
Two	3.80	12.77	2.76	0.81	0.75
Three	2.84	9.58	2.20	0.68	0.75
Four	2.44	8.26	3.03	0.94	0.78

**Table 2:** Means, standard deviations, factor loadings and communalities in the initial 30-items version of the Questionnaire on Academic Stress in Secondary Education (QASSE).

Item	M	DS	Factorial Saturations				Communality
			1	2	3	4	
1. Taking exams	3.68	0.99	0.768	−0.009	0.125	0.182	0.639
2. Presentations of work in class	2.94	1.11	0.362	0.243	0.125	0.041	0.207
3. Intervening in class (e.g., asking questions, participating in debates)	2.14	0.97	0.133	0.587	0.042	0.149	0.386
4. Dealing with the teacher outside of class (e.g., in homeroom, office visits)	2.10	1.07	0.094	0.565	0.162	0.065	0.359
5. Academic overload (having too many exams and tasks to do)	3.95	1.02	0.769	0.045	0.140	0.106	0.624
6. Lack of time to fulfill all the activities we are asked to do	3.57	1.13	0.759	0.034	0.108	0.045	0.591
7. Competitiveness among classmates	2.07	1.07	0.136	0.599	−0.109	0.260	0.457
8. Doing tasks that involve looking for information and writing	2.50	1.01	0.515	0.409	−0.037	0.120	0.435
9. The task of studying (e.g., meeting established schedules, level of effort)	3.28	1.07	0.726	0.106	0.107	0.069	0.555
10. Working with classmates on tasks in class	2.19	0.97	0.271	0.613	−0.029	0.124	0.466
11. Problems or conflicts with teachers	2.14	1.22	0.031	0.492	0.478	−0.076	0.478
12. Problems or conflicts with classmates	1.95	1.09	−0.032	0.573	0.299	−0.035	0.420
13. Being able to attend all the classes	2.01	1.18	0.103	0.446	0.406	0.050	0.326
14. Too much responsibility to fulfill my obligations	3.05	1.11	0.667	0.254	0.180	0.152	0.565
15. Obtaining high grades in different subjects	3.37	1.19	0.611	0.015	−0.001	0.409	0.540
16. Future academic and professional perspectives	3.29	1.22	0.386	0.077	0.092	0.584	0.650
17. Choosing subjects in the coming courses	2.49	1.16	0.157	0.241	0.109	0.711	0.600
18. Getting or keeping a grant to study	2.94	1.35	0.170	0.194	0.164	0.750	0.656
19. Finishing 10th grade (or 12th grade or Vocational Education) in the stipulated time periods	3.20	1.37	0.320	0.187	0.374	0.511	0.539
20. Family pressure to obtain good grades	3.21	1.36	0.244	0.076	0.661	0.256	0.569
21. Lack of support from my teachers	2.38	1.16	0.173	0.436	0.443	0.158	0.442
22. Keeping up with the academic activities and tasks	2.83	1.02	0.683	0.226	0.177	0.085	0.557
23. My relationships with my classmates	2.08	1.16	0.013	0.719	0.114	0.018	0.530
24. Doing things well in all the subjects in the course	3.21	1.07	0.694	0.160	0.046	0.217	0.557
25. Family discussions and conflicts caused by my studies	2.82	1.39	0.135	0.140	0.770	0.028	0.631
26. Making leisure time and academic work compatible	3.14	1.12	0.687	0.074	0.198	0.037	0.517
27. Teachers’ pressure about my work and behavior	2.73	1.18	0.275	0.353	0.483	0.095	0.442
28. The fact that my parents are always on top of me (e.g., whether I do my homework and activities, my grades, …)	2.78	1.45	0.094	0.129	0.755	0.141	0.616
29. Doing poorly on an exam.	3.93	0.99	0.503	0.040	0.001	0.287	0.338
30. The fact that my classmates think I’m not a good student	2.42	1.31	0.104	0.460	0.162	0.235	0.304

**Table 3:** Goodness-of-fit indexes for the confirmatory factor analysis (CFA) models.

Models	χ^2^	df	p	CFI	RMSEA	90% CI for the RMSEA
One-dimensional (M1)	2557.5	252	<0.001	0.699	0.121	0.117–0.126
Four oblique factors (M2)	965.5	246	<0.001	0.906	0.069	0.064–0.073
Second order factor (M3)	991.6	248	<0.001	0.903	0.069	0.065–0.074
Four oblique first-order factors and a cross-loading of item 27 (M2r#)	856.1	245	<0.001	0.920	0.063	0.059–0.068
Second order factor + a cross-loading of item 27 (M3r#)	847.1	247	<0.001	0.922	0.063	0.058–0.067

*Note*: χ^2^ = chi-square; *df* = degrees of freedom; CFI = Comparative fit index; RMSEA = Root mean squared error of approximation; 90% CI = Confidence interval for RMSEA. M2r# and M3r# models are the same as M2 and M3 respectively, a cross-loading of item 27.

**Table 4:** Means (M), Standard Deviations (SD), Skewness (Sk), Kurtosis (Ku), Correlation matrix and Internal consistency (Cronbach’s alphas on the diagonal) of latent variables.

Latent Variables	M	SD	Sk	Ku	Correlation matrix						
					1	2	3	4	5	6	7
1. Academic Overload	3.39	0.74	−0.40	−0.03	(0.86)						
2. Interactions with Classmates	2.13	0.68	0.43	−0.27	0.50 ***	(0.73)					
3. Family Pressure	2.81	1.05	0.12	−0.96	0.49 ***	0.36 ***	(0.72)				
4. Future Perspectives	3.02	0.97	−0.13	−0.76	0.62 ***	0.45 ***	0.45 ***	(0.75)			
5. Academic stress (second order factor)	2.86	0.61	−0.19	0.05	0.82 ***	0.61 ***	0.60 ***	0.75 ***	(0.88)		
6. Well-being (GHQ-12)	2.39	0.58	0.29	−0.52	0.59 ***	0.44 ***	0.43 ***	0.54 ***	0.69 ***	(0.72)	
7. Somatic complaints (LQS)	1.74	0.42	0.40	−0.57	0.46 ***	0.34 ***	0.33 ***	0.42 ***	0.72 ***	0.55 ***	(0.84)

*Note*: *** *p* < 0.001.

**Table 5:** Main effects and interaction effects of gender and educational level variables on the dimensions of academic stress.

Dependent Variable	Gender				Educational Level				Gender x Educational Level			
	*F*	gl	*p*	*η* ^2^	*F*	gl	*p*	*η* ^2^	*F*	gl	*p*	*η* ^2^
AO	19.81	1	0.001	0.02	8.28	5	0.001	0.05	5.72	5	0.001	0.03
IC	1.62	1	0.20	0.00	1.92	5	0.09	0.01	2.32	5	0.05	0.01
FP	1.25	1	0.26	0.00	10.18	5	0.001	0.06	1.61	5	0.16	0.01
FP	4.96	1	0.001	0.03	4.96	5	0.001	0.03	2.68	5	0.02	0.02
AS_SO	2.93	1	0.02	0.01	2.93	5	0.02	0.02	3.00	5	0.01	0.02

*Note*: AO = Academic Overload; IC = Interaction with classmates; FP = Family Pressure; FP = Future Perspectives; AS_SO = Academic Stress second order factor.
